# SSX2IP promotes metastasis and chemotherapeutic resistance of hepatocellular carcinoma

**DOI:** 10.1186/1479-5876-11-52

**Published:** 2013-03-01

**Authors:** Pu Li, Ying Lin, Yu Zhang, Zhenggang Zhu, Keke Huo

**Affiliations:** 1 State Key Laboratory of Genetic Engineering, Institute of Genetics, School of Life Sciences, Fudan University, 220 Handan Rd, Shanghai 200433, People’s Republic of China; 2 Shanghai Key Laboratory of Gastric Neoplasms, Department of Surgery, Shanghai Institute of Digestive Surgery, Ruijin Hospital, School of Medicine, Shanghai Jiao Tong University, 197 Rui Jin Er Rd, Shanghai 200025, People’s Republic of China; 3 Department of Developmental Biology, Memorial Sloan-Kettering Cancer Center, Sloan-Kettering Institute, RRL8411275 York Ave, New York, NY10065, USA

**Keywords:** SSX2IP, Hepatocellular carcinoma, Metastasis, Chemotherapeutic resistance

## Abstract

**Background:**

Synovial sarcoma, X breakpoint 2 interacting protein (SSX2IP), which has been identified as an acute myeloid leukemia associated antigen, is a potential target for leukemia immunotherapy. In rodents, its homologous gene, ADIP, plays an important role in the regulation of cell adhesion and migration, underlying its potential role in promoting metastasis of other cancers.

**Methods:**

To investigate the correlation between the expression level of SSX2IP and the clinicopathologic factors of hepatocellular carcinoma (HCC), 53 cases were studied by qPCR and statisted. To directly testing SSX2IP’s contribution to HCC in animal models, 45 nude mice were enrolled in peritoneal spreading and liver metastasis models. For the migration and invasion assays, cell culture experiments were performed using QCM^TM^ 24-Well Colorimetric Migration Assay Kit and Cell Invasion Assay Kit (Millipore). Moreover we examined the influence of SSX2IP overexpression on the chemosensitivity of hepatocellular carcinoma cells to two most common chemotherapy drugs (5-Fu and CDDP) using Cell counting kit-8 (CCK-8). The chemotherapeutic drugs sensitivity was evaluated by IC50 parameter.

**Results:**

Statistical analysis of clinical cases revealed that the SSX2IP high expression group had inclinations towards larger tumor size, more tumor thrombus and shorter survival period, implying a strong correlation between the expression level of SSX2IP and HCC tumorigenesis. Consistently in abdominal cavity metastasis and liver metastasis models of immune-deficient mice, SSX2IP was able to promote the metastasis of hepatoma cells. At the cytological level, SSX2IP stimulates the wound healing, metastasis and invasion of hepatoma cells, and reduces the sensitivity of hepatoma cells to 5-Fu and CDDP.

**Conclusions:**

Our results showed that SSX2IP promotes the development and metastasis of hepatocellular carcinoma and contributes to the drug resistance of hepatoma cells, suggesting that SSX2IP is expected to become a new diagnostic and prognostic marker and a new target of the treatment of hepatocellular carcinoma.

## Background

Hepatocellular carcinoma (HCC) is one of the most common malignant tumors, ranking the fifth of the global malignant tumor incidence and the third of cancer related cause of death [1,2]. In China, hepatocellular carcinoma incidence ranks second only to lung cancer and counts for about half of the global number, thus people’s health and life quality have been seriously affected. As the disease symptom is not obvious, early diagnosis is difficult; most of the patients have advanced cancer at the time of diagnosis, and the disease is easy to relapse even if the lesions were resected. Although chemotherapy is an important method in combined treatment of hepatocellular carcinoma, its effect becomes less unsatisfactory as hepatoma cells from advanced cancer often develop resistance to chemotherapeutic drugs. Therefore, identifying molecular markers suitable for early diagnosis and new therapeutic targets for treatment will greatly benefit the many HCC patients around the world.

The *SSX2IP* gene is located on chromosome 1p22.3, spanning 46 kb region and containing 14 exons [3]. The locus has a higher frequency of deletions, amplifications and translocations [4-6]. SSX2IP was originally identified through its interaction with SSX2, a transcriptional repressor [7], and was suggested to regulate the role of SSX2 in testicular tissue and malignant cells [8]. Previous studies indicated that SSX2IP is an acute myeloid leukemia-associated antigen, as analysis of clinical cases showed that the expression of SSX2IP was identified in the serum of 33% patients with acute myeloid leukaemia (AML), but not in healthy volunteers [9]. SSX2IP expression was most apparent on the surface of myeloid leukaemia cells in mitosis [10,11]. Several studies further strengthened the argument that SSX2IP was an acute myeloid leukemia-associated antigen and a potential immunotherapy target for leukemia [12-14]. So far, the role of SSX2IP in other human tumors remains elusive.

Afadin DIL domain-interacting protein (*ADIP*) is the homologous gene of *SSX2IP* in rodents, and the amino acid sequences of ADIP in mouse and rat show 88% and 87% identity with SSX2IP respectively. Cytologically, ADIP localizes at cell-cell adherens junctions and promotes cell migration by activating Rac protein through Vav2 [15,16]. All of the above characteristics are closely related to the adhesion capacity and metastatic characteristics. Given the functionality prompts of SSX2IP and its homologue gene ADIP, we decided to explore the role of SSX2IP in the development and progression of hepatocellular carcinoma.

## Methods

### Cell culture and transfection

Human hepatocellular carcinoma cell lines SMMC-7721 and BEL-7402 were preserved in our institute. Briefly, cells were grown in DMEM supplemented with 10% fetal calf serum. Cells were maintained at 37°C with 5% CO_2_. SSX2IP was constructed into pEGFP-C1 eukaryotic expression vector. We obtained stably transfected clones by G418 selection (Promega, 800 μg/ml). A stable transfectant of the pEGFP-C1 empty vector was used as a control.

### Migration, invasion and scratch wound healing assays

For the cell migration assays, cell culture was performed using QCM^TM^ 24-Well Colorimetric Migration Assay Kit (Millipore) according to the manufacturer’s instructions. For the invasion assay, Cell Invasion Assay Kit (Millipore) was used according to the manufacture’s instructions. Cells (1 × 10^5^) in 300 μl serum-free medium were added to the upper chambers and cultured for 48 h. Non-migrating or non-invading cells were removed with cottons swabs, Cells that migrated or invaded to the bottom of the membrane were stained with the cell stain buffer provided in the assay kit and counted under microscope and photographed. Three independent experiments were performed for the same conditions. For the scratch wound healing assay, cells were cultured in serum-free medium for 24 h and wounded with pipette tips. Fresh medium was replaced. The wound closing procedure was observed for 48 h, and photographs were taken every 24 h.

### Nude mice study

Both peritoneal spreading and liver metastasis models were observed. 4-week-old male BALB/C nude mice were purchased from the Institute of Zoology, Chinese Academy of Sciences of Shanghai. All experiments were performed in accordance with the official recommendations of the Chinese animal community. In peritoneal spreading experiment, 5 mice were enrolled in each group. 1 × 10^6^ cells of BEL-7402, BEL-7402^Vector^ and BEL-7402^SSX2IP^ were injected into each mouse. In liver metastasis study, 10 mice were enrolled in each group. 1 × 10^6^ cells of BEL-7402, BEL-7402^Vector^ and BEL-7402^SSX2IP^ were injected into caudal vena. 6 weeks after intraperitoneal injection, mice were killed by cervical decapitation, the abdominal masses were excised and fixed with buffered formalin for further morphological analysis. 10 weeks after caudal vena injection, mice were killed by cervical decapitation. The liver of mice was examined carefully and the metastatic masses were counted.

### Chemotherapeutic drugs sensitivity analysis by cell counting kit-8

Hepatocellular carcinoma cells (3 × 10^3^ cells/well) were seeded in 96-well plates and treated with different chemotherapeutics 5-fluorouracil (5-Fu) and Cis-diamminedichloroplatinum (CDDP) in different concentrations for 72 h. Cell Counting Kit-8 (CCK-8, Dojindo, Japan) was added into each well. After 4 h incubation at 37°C, the coloring reaction was quantified by an automatic plate reader (BIO.TEK, USA). Growth inhibitory effects were expressed as cell viability curve (OriginPro 8.5.1, USA). The chemotherapeutic drugs sensitivity was evaluated by IC50 parameter (inhibitory concentration of 50% cells).

### Patients

HCC tissues were collected from patients at Zhongshan Hospital, Fudan University, after obtaining the subjects’ informed consent and with institutional review board approval of the hospital.

### mRNA expression analysis

Total RNA was extracted using Trizol solution (Invitrogen). Reverse transcription was performed according to the manufacturer’s instructions (Promega). qPCR was performed in triplicate using the SYBR Green Mastermix on the ABI Prism 7900 Sequence Detection System (Applied Biosystems). qPCR primers used as follows: SSX2IP: 5’-CCGGGGAACTAAGCAGAGAGA-3’(Forward), 5’-GTTCATGGTCTTGTCGTGAGAT-3’(Reverse); GAPDH: 5’-GCACCGTCAAGGCTGAGAAC-3’(Forward), 5’-ATGGTGGTGAAGACGCCAGT-3’ (Reverse).

### Statistics

Data were shown as mean ± SD. Statistically significant differences were analyzed using Student *t* test, when there were only two groups, multiple comparisons were performed by one-way analysis of variance. For survival analysis, Kaplan-Meier method was used. Log rank test was used to analyze differences between statistical significances. All statistical analyses were performed using the SPSS 13.0 software (SPSS Inc, Chicago, IL, USA). *P* < 0.05 was considered statistically significant.

## Results

### The expression levels of SSX2IP in HCC patients were significantly correlated with tumor size, tumor thrombosis and survival time

**Figure 1:**
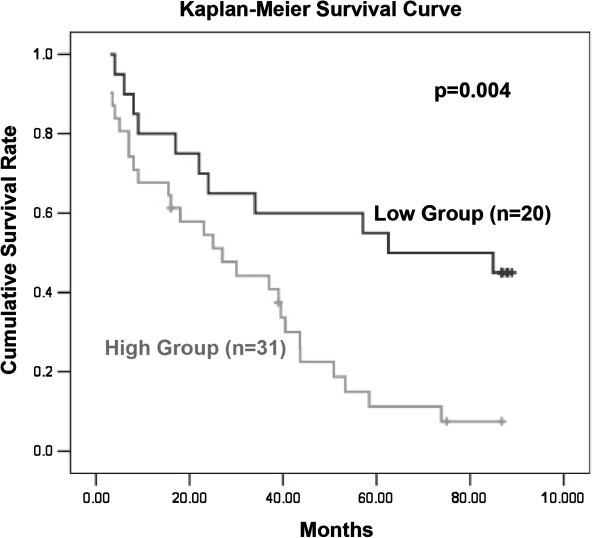
**Kaplan-Meier survival analysis of SSX2IP expression level in 51 HCC patients.** The survival time in SSX2IP high expression group (n = 31) was shorter than in the low expression group (n = 20), the difference was significant (*P* = 0.004).

To examine the correlation between the expression level of SSX2IP and clinicopathologic factors in HCC, 53 cases were statistically analysed. The clinical and pathologic characteristics of these cases were shown in Table 1. Among them, 32 samples showed a ~2-fold up-regulation of SSX2IP compared with the matched non-tumor controls. These samples were classified as the high expression group, and the other 21 samples were classified as the low expression group. Importantly, the SSX2IP high expression group had inclinations towards larger tumor size and more tumor thrombus (*P* < 0.05). However, there were no significant relationships with other clinicopathologic factors. Notably, 51 out of 53 patients were re-interviewed with a re-interview rate of 96%, Kaplan-Meier survival analysis showed the patient survival time in SSX2IP high expression group (n = 31) was significantly shorter than in the low expression group (n = 20) (*P* = 0.004, Figure 1). Taken together, our patient cases studies suggest that SSX2IP may have a previously unrecognized role in promoting hepatocellular carcinoma.

**Table 1:** Relationship between SSX2IP expression level and clinicopathologic variables in 53 HCC patients

**Clinicopathologic parameters**	**SSX2IP expression level**		***P***
	**Low (n = 21)**	**High (n = 32)**	
Gender			
Female	3	6	0.961
Male	18	26	
Age			
>50	11	19	0.615
≤50	10	13	
Tumor size(cm)			
>5	8	21	0.049
≤5	13	11	
Tumor encapsulation			
Complete	13	16	0.394
Incomplete	8	16	
Tumor differentiation			
I II	11	24	0.089
III IV	10	8	
Tumor number			
Single	17	27	1.000
Multiple	4	5	
Lymph node invasion			
No	20	29	0.928
Yes	1	3	
Tumor thrombus			
No	18	18	0.025
Yes	3	14	
AFP(ng/mL)			
≤400	5	12	0.296
>400	16	20	

### SSX2IP promotes peritoneal spreading and liver metastasis of hepatocellular carcinoma cells in nude mice

**Figure 2:**
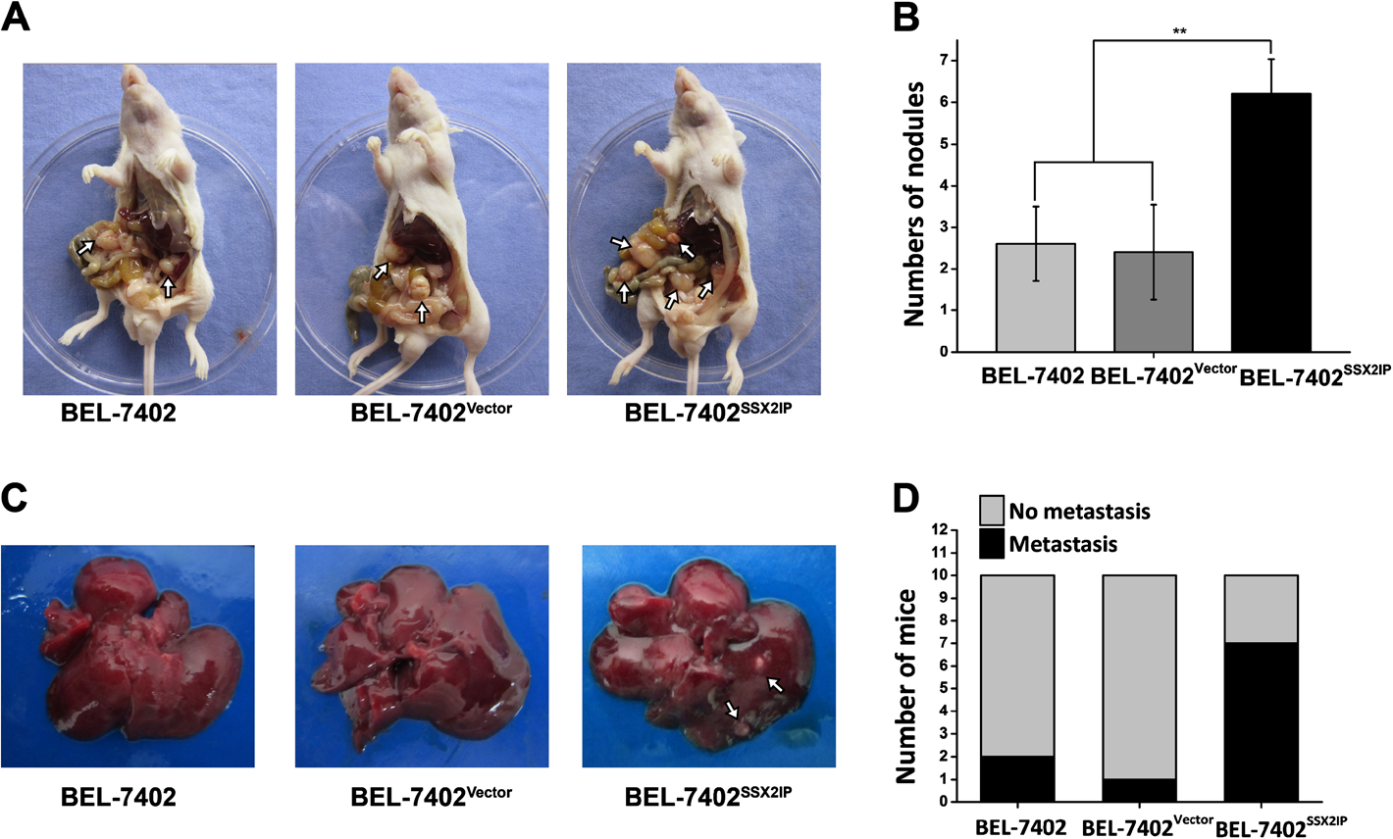
**Effect of enforcing SSX2IP on peritoneal spreading and liver metastasis.** 1 × 10^6^ cells per mouse were injected into peritoneal cavity or caudal vena. **A**. BEL-7402, BEL-7402^Vector^ and BEL-7402^SSX2IP^, the three cell lines, were injected into the peritoneal cavity of nude mice. 6 weeks later, we took the photographs of the peritoneal nodles. **B**. The peritoneal nodles were significantly enforced in SSX2IP overexpression group, compared with the parental cells and empty vector-transfected cells groups(6.2 ± 0.84 vs 2.6 ± 0.89, 2.4 ± 1.14, **P < 0.001). **C**. BEL-7402, BEL-7402^Vector^ and BEL-7402^SSX2IP^, the three cell lines, were injected into the caudal vena of nude mice. 10 weeks later, we took the photographs of the nude mice liver. **D**. Statistical proportion of liver metastasis mice. In SSX2IP overexpression group, 7 out of 10 mice show tumorigenicity in liver, which is significantly higher than that in parental and empty vector-transfected groups (2/10 and 1/10, *P* = 0.023).

To directly test the contribution of SSX2IP in HCC, we carried out peritoneal spreading and liver metastatic assays in mice models. A total of 45 nude mice were enrolled in these experiments, among which, 15 mice were used for peritoneal spreading assay and 30 for liver metastatic assay. The results were shown in Figure 2 and summarized in Table 2 and Table 3. Strikingly, the peritoneal nodules were significantly enforced in SSX2IP over expression group compared with the parental and empty vector-transfected groups (6.2 ± 0.84 vs 2.6 ± 0.89, 2.4 ± 1.14, **P < 0.001, Figure 2A-B and Table 2). The tumorigenicity of liver metastasis was also observed. In SSX2IP overexpression group, 7 out of 10 mice showed tumorigenicity in liver, which is significantly higher than that in the parental group (2/10) and the empty vector-transfected group (1/10) (*P* = 0.023, Figure 2C-D and Table 3). The results clearly reveal a promoting role of SSX2IP in hepatocellular carcinoma development.

**Table 2:** Animal experiments for peritoneal spreading

**Animal models**	**Cells**	**Cell numbers of injection**	**Time**	**Tumorigenicity**	***P***
Peritoneal spreading	Bel-7402	1 × 10^6^	6 weeks	2.6 ± 0.89	*P* < 0.001
	Bel-7402^Vector^	1 × 10^6^	6 weeks	2.4 ± 1.14	
	Bel-7402^SSX2IP^	1 × 10^6^	6 weeks	6.2 ± 0.84	

**Table 3:** Animal experiments for liver metastasis

**Animal models**	**Cells**	**Cell numbers of injection**	**Time**	**No. of metastasis mice/No. of mice injected**	***P***
Liver metastasis	Bel-7402	1 × 10^6^	10 weeks	2/10	
	Bel-7402^Vector^	1 × 10^6^	10 weeks	1/10	*P* = 0.023
	Bel-7402^SSX2IP^	1 × 10^6^	10 weeks	7/10	

### SSX2IP enhances scratch wound healing ability of hepatocellular carcinoma cells

**Figure 3:**
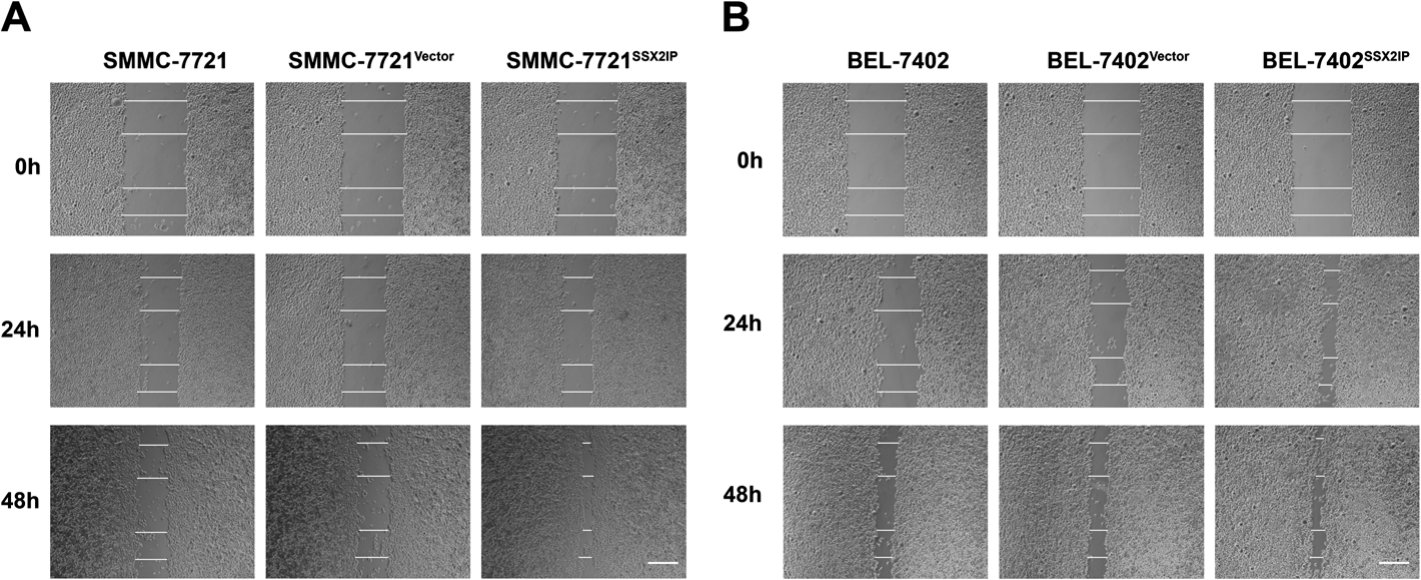
**SSX2IP enhances scratch wound healing ability of hepatocellular carcinoma cells. A**. Movement ability of SMMC-7721, SMMC-7721^Vector^ and SMMC-7721^SSX2IP^ cell lines was detected by scratch wound healing assays. **B**. Movement ability of BEL-7402, BEL-7402^Vector^ and BEL-7402^SSX2IP^ cell lines was detected by scratch wound healing assays. By wound distance analysis, the movement ability of HCC cells was enhanced by SSX2IP.

We next examined the cytological effect of SSX2IP on movement ability of HCC cells by scratch healing assay. Consistent with our results from mice models, SSX2IP overexpression cell lines SMMC-7721^SSX2IP^ and BEL-7402^SSX2IP^ largely sealed the wound 48 h after scratching, in contrast to the parental cells and empty vector-transfected cells which were significantly less efficient in healing, as indicated by the mean wound distances (SMMC-7721 cell line groups: 170.83 ± 36.96 μm vs 516.67 ± 25.57 μm and 525.00 ± 31.92 μm, **P < 0.001. BEL-7402 cell line groups: 183.33 ± 49.07 μm vs 312.50 ± 25.00 μm and 329.17 ± 15.96 μm, **P < 0.001, Figure 3).

### SSX2IP promotes migration and invasion of hepatocellular carcinoma cells

**Figure 4:**
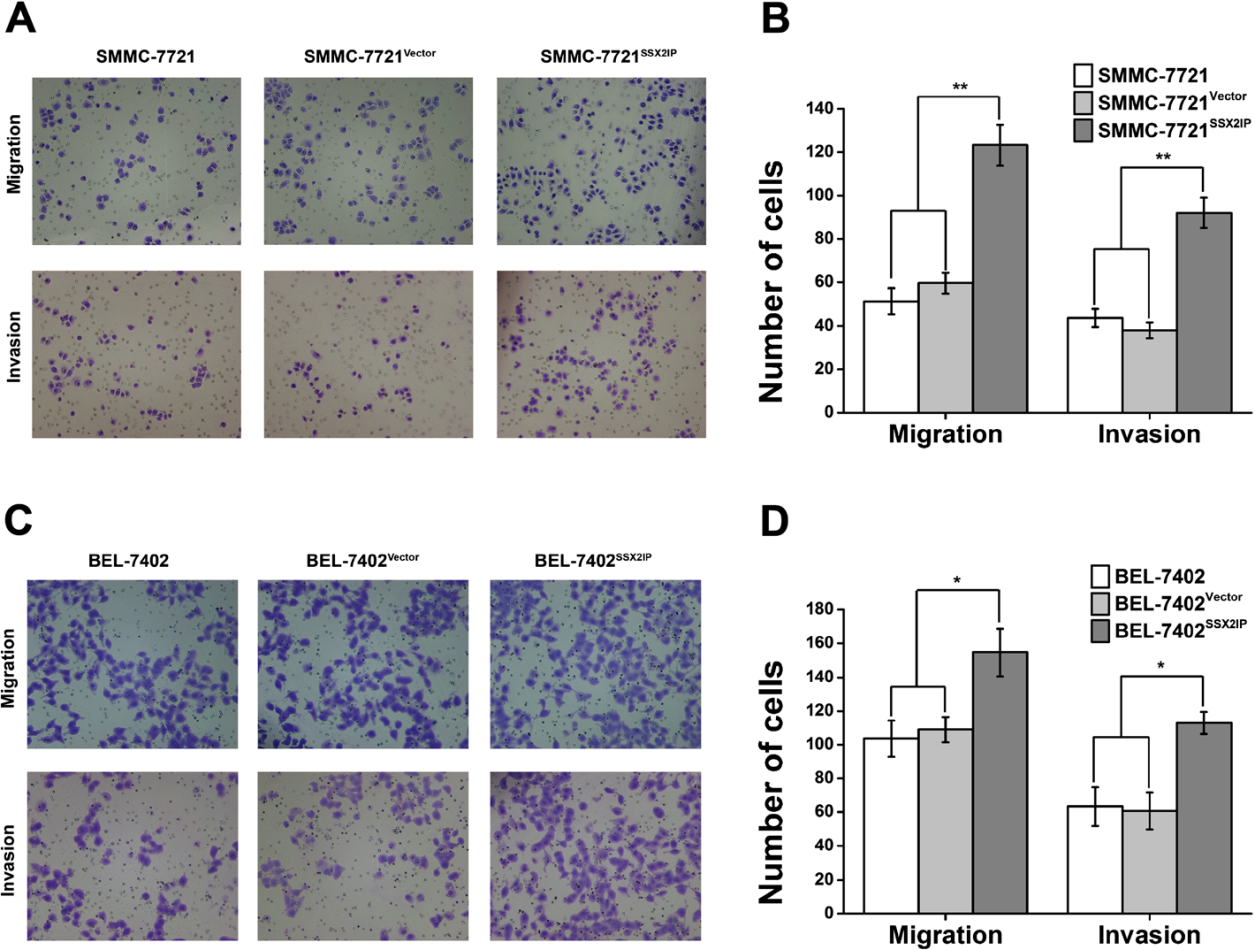
**SSX2IP promotes migration and invasion of hepatocellular carcinoma cells. A**. Representative photographs of migratory or invasive SMMC-7721 cells on the membrane (magnification, 100×). **B**. Average number of migratory or invasive SMMC-7721 cells. (**P < 0.001). **C**. Representative photographs of migratory or invasive BEL-7402 cells on the membrane (magnification, 100×). **D**. Average number of migratory or invasive BEL-7402 cells (*P < 0.01). The data represent the mean ± s.d. of three independent experiments.

In order to further assess the influence of SSX2IP on HCC cells, we employed cell migration and invasion assays to determine these two key factors of malignant progression and metastasis upon SSX2IP overexpression. As shown in result, overexpression of SSX2IP led to significantly enhanced migration and invasion of both SMMC-7721 and BEL-7402 (Figure 4). Number of migratory SMMC-7721^SSX2IP^ cell line is significantly higher than the other two control groups (123.33 ± 9.45 vs 59.67 ± 4.73 and 51.33 ± 6.03), and number of invasive SMMC-7721^SSX2IP^ cell line is significantly higher compared to the control groups (92.00 ± 7.00 vs 43.67 ± 4.16 and 38.00 ± 3.61, **P < 0.001, Figure 4A-B). Similarly, number of migratory BEL-7402^SSX2IP^ cell line is significantly higher than the other two control groups (154.67 ± 14.05 vs 103.67 ± 10.70 and 109.00 ±7.55), and number of invasive BEL-7402^SSX2IP^ cell line is significantly higher compared to the control groups (113.00 ± 6.56 vs 63.33 ± 11.50 and 60.67 ± 11.02, *P < 0.01, Figure 4C-D). These results displayed an enhanced effect of SSX2IP on hepatocellular carcinoma cells migration and invasion.

### SSX2IP reduces the sensitivity of hepatocellular carcinoma cells to chemotherapeutic drugs

**Figure 5:**
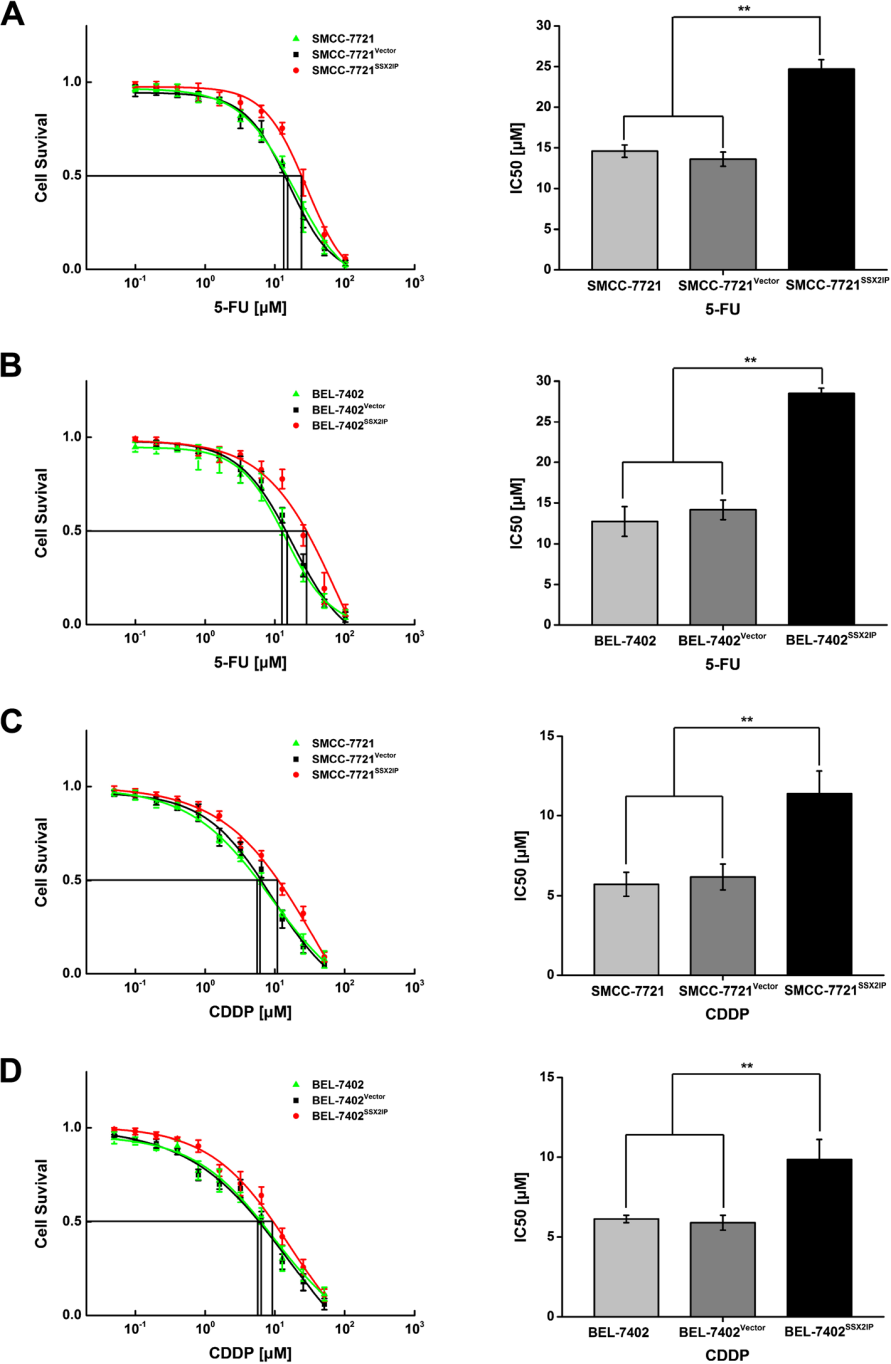
**SSX2IP reduces the sensitivity of hepatocellular carcinoma cells to chemotherapeutic drugs. A**. Dose–response curves and IC50 values for 5-Fu between SMMC-7721, SMMC-7721^Vector^ and SMMC-7721^SSX2IP^ cell lines. **B**. Dose–response curves and IC50 values for 5-Fu between BEL-7402, BEL-7402^Vector^ and BEL-7402^SSX2IP^ cell lines. **C**. Dose–response curves and IC50 values for CDDP between SMMC-7721, SMMC-7721^Vector^ and SMMC-7721^SSX2IP^ cell lines. **D**. Dose–response curves and IC50 values for CDDP between BEL-7402, BEL-7402^Vector^ and BEL-7402^SSX2IP^ cell lines. The IC50 values of SMMC-7721^SSX2IP^ and BEL-7402^SSX2IP^ cell lines for 5-Fu and CDDP are higher, compared to those in control groups (**P < 0.001).

The positive effect of SSX2IP on HCC cells’ metastatic status prompted us to speculate that it may also contribute to the drug resistance of HCC cells. To test this idea, we assayed chemosensitivity to the two most common chemotherapy drugs, 5-Fu and CDDP, to compare SSX2IP overexpression groups (SMMC-7721^SSX2IP^ and BEL-7402^SSX2IP^) and the according control groups (SMMC-7721, SMMC-7721^Vector^, BEL-7402 and BEL-7402^Vector^). The results showed that the IC50s of both 5-Fu and CDDP were significantly increased by the overexpression of SSX2IP in both SMMC-7721 and BEL-7402 lines (Figure 5). SMMC-7721^SSX2IP^ and BEL-7402^SSX2IP^ for 5-Fu was 24.68 ± 1.17 μM and 28.52 ± 0.65 μM, respectively, while the IC50s of SMMC-7721, SMMC-7721^Vector^, BEL-7402 and BEL-7402^Vector^ for 5-Fu were 14.59 ± 0.77 μM, 13.60 ± 0.88 μM, 12.73 ± 1.81 μM and 14.16 ± 1.20 μM (**P < 0.001, Figure 5A-B). The IC50s of SMMC-7721^SSX2IP^ and BEL-7402^SSX2IP^ for CDDP were 11.38 ± 1.42 μM and 9.86 ± 1.24 μM, respectively, while the IC50s of SMMC-7721, SMMC-7721^Vector^, BEL-7402 and BEL-7402^Vector^ for CDDP were 5.72 ± 0.75 μM, 6.18 ± 0.81 μM, 6.13 ± 0.24 μM and 5.90 ± 0.47 μM, respectively (**P < 0.001, Figure 5C-D). We could concluded that SSX2IP contributes to reduce the sensitivity of HCC cells to chemotherapeutic drugs.

## Discussion

Statistical analysis of pathological features and postoperative survival of clinical cases of hepatocellular carcinoma patients demonstrated that tumor with SSX2IP high expression had inclinations towards larger tumor size and more tumor thrombus. Patients with high SSX2IP expression experienced significantly shorter periods of postoperative survival. These data indicated great significance of SSX2IP in the clinical research of hepatocellular carcinoma.

Metastatic ability of hepatoma cells is the key factor for HCC development. In intraperitoneal injection and tail vein injection experiments in nude mice, over-expression of SSX2IP can promote intraperitoneal spreading and formation of liver metastases. In vitro studies, scratch wound healing assay and transwells migration assay have been widely used for studying the motility of tumor cells to reflect their ability to penetrate extracellular matrix and to migrate. Hepatoma cells with high expression of SSX2IP were shown a very strong mobility, and the number of cells which had penetrate the filter membrane in this group was significantly higher than in the parental and empty vector-transfected groups. These results suggest that SSX2IP could promote the migratory and invasive ability of hepatoma cells.

The dynamic changes of cell adhesion characteristics and the reorganization of cytoskeletal play important roles in the process of tumor invasion and metastasis [17,18]. *ADIP*, the homologous gene of *SXX2IP* in rodents, localizes at cell-cell adherent junctions and binds to α-actinin, a kind of F-actin-bundling protein. ADIP may play a key role in the organization of the cytoskeleton at cell-cell adherent junctions and may connect the nectin-afadin and E-cadherin-catenin systems [15]. These systems have been shown to maintain the epithelial to mesenchymal transition (EMT) and mesenchymal-epithelial transition (MET), and studies of a variety of tumors have revealed that this conversion characteristics of the tumor cells is an important reason for the tumor metastasis and recurrence [19-21]. Therefore, it is suggested that SSX2IP may regulate the EMT-MET by affecting E-cadherin-catenin systems and thereby promote the invasion and metastasis of tumor cells.

On the other hand, ADIP has been shown to promote cell movement by regulating the activation of Rac1 [16]. Rac1 belongs to the Rho family and the Ras superfamily of small GTP-binding proteins, and it is an important regulator which connects membrane surface receptors and actin cytoskeleton and plays a role of molecular switch. Members of Rho family regulate the actin assembly and affect cell movement, tumor invasion and migration [22]. The guanine nucleotide exchange factor specific to Rac1 can regulate cytoskeleton reorganization, affect cell polarity and promote cell motility and migration by promoting the conversion of Rac-GDP (inactivation) to Rac1-GTP (activation) [23]. As our study indicated a role of SSX2IP in promoting the mobility of HCC cells, we speculated that SSX2IP may do so via activating Rac1. We are actively investigating the influence of SSX2IP on Rac1 activation.

Chemotherapy plays an irreplaceable role in the clinical treatment of HCC [24]. However, resistance of hepatoma cells to chemotherapy drugs often makes chemotherapy unsatisfactory [25]. High expression of some resistance related genes in tumor cells is the underlying cause of drug resistance that eventually leads to ineffective treatment. Our data showed SSX2IP reduced the sensitivity of hepatoma cells to 5-Fu and CDDP and significantly increased IC50, which indicated that SSX2IP may be a resistance related gene with the ability to promote the resistance of hepatoma cells to chemotherapeutic drug. Although the exact function of SSX2IP in drug resistance is still unknown, clues can be taken from its rodent homologue ADIP, which directly binds to β’-COP to participate in the vesicle trafficking from the Golgi apparatus to the endoplasmic reticulum [26]. As is well known, many resistance genes encode transporter proteins, such as ABCG2 in the ABC protein family [27,28]. We speculate that SSX2IP is likely to be a player in the intracellular transportation system.

Studies of SSX2IP in tumor-related fields have been focused on leukemia in the past, while little is known about its role in oncology of other types of cancers. Our results suggest that SSX2IP can significantly promote the motility, invasion and migration of hepatoma cells and improve their resistance to chemotherapeutic drugs. Importantly, since SSX2IP has been identified as an acute myeloid leukaemia-associated antigen and a potential immunotherapy target for leukemia, this study has expanded the usage of those SSX2IP related applications to HCC. Further in-depth exploration of the molecular mechanisms of SSX2IP in promoting the migration, invasion and drug resistance of HCC will be needed to clarify the role of SSX2IP in HCC development and to provide theoretical basis for future clinical applications.

## Conclusions

Our studies showed that SSX2IP can promote the development and metastasis of hepatocellular carcinoma. It reduces the sensitivity of hepatocellular carcinoma cells to chemotherapeutic drugs and could be a resistance-associated gene. All the above, our findings suggest that SSX2IP may serve as a new target for clinical diagnosis and treatment of hepatocellular carcinoma.

## Abbreviations

HCC: Hepatocellular carcinoma; SSX2IP: Synovial sarcoma X breakpoint 2 interacting protein; ADIP: Afadin DIL domain-interacting protein; AFP: α-fetoprotein; 5-Fu: 5-fluorouracil; CDDP: Cis-diamminedichloroplatimum; IC50: Half maximal (50%) inhibitory concentration (IC) of a substance.

## Competing interests

The authors declare that they have no competing interests.

## Authors’ contributions

KKH and ZGZ conceived the study design, participated in its design and in the acquisition of data. PL carried out the experiments, participated in the acquisition of data, analysis and interpretation, drafted the manuscript. YL has been involved in analyzing the data and drafting the manuscript. YZ helped to draft and revise the manuscript. All authors read and approved the final manuscript.
